# HIV-1-Infected and Immune-Activated Macrophages Induce Astrocytic Differentiation of Human Cortical Neural Progenitor Cells via the STAT3 Pathway

**DOI:** 10.1371/journal.pone.0019439

**Published:** 2011-05-27

**Authors:** Hui Peng, Lijun Sun, Beibei Jia, Xiqian Lan, Bing Zhu, Yumei Wu, Jialin Zheng

**Affiliations:** 1 Laboratory of Neuroimmunology and Regenerative Therapy, University of Nebraska Medical Center, Omaha, Nebraska, United States of America; 2 Departments of Pharmacology and Experimental Neuroscience, University of Nebraska Medical Center, Omaha, Nebraska, United States of America; 3 Departments of Pathology and Microbiology, University of Nebraska Medical Center, Omaha, Nebraska, United States of America; University of Leuven, Rega Institute, Belgium

## Abstract

Diminished adult neurogenesis is considered a potential mechanism in the pathogenesis of HIV-1-associated dementia (HAD). In HAD, HIV-1-infected and immune-activated brain mononuclear phagocytes (MP; perivascular macrophages and microglia) drive central nervous system (CNS) inflammation and may alter normal neurogenesis. We previously demonstrated HIV-1-infected and lipopolysaccharide (LPS) activated monocyte-derived macrophages (MDM) inhibit human neural progenitor cell (NPC) neurogenesis, while enhancing astrogliogenesis through the secretion of the inflammatory cytokines such as tumor necrosis factor-alpha (TNF-α), *in vitro* and *in vivo*. Here we further test the hypothesis that HIV-1-infected/activated MDM promote NPC astrogliogenesis via activation of the transcription factor signal transducer and activator of transcription 3 (STAT3), a critical factor for astrogliogenesis. Our results show that LPS-activated MDM-conditioned medium (LPS-MCM) and HIV-infected/LPS-activated MDM-conditioned medium (LPS+HIV-MCM) induced Janus kinase 1 (Jak1) and STAT3 activation. Induction of the Jak-STAT3 activation correlated with increased glia fibrillary acidic protein (GFAP) expression, demonstrating an induction of astrogliogenesis. Moreover, STAT3-targeting siRNA (siSTAT3) decreased MCM-induced STAT3 activation and NPC astrogliogenesis. Furthermore, inflammatory cytokines (including IL-6, IL-1β and TNF-α) produced by LPS-activated and/or HIV-1-infected MDM may contribute to MCM-induced STAT3 activation and astrocytic differentiation. These observations were confirmed in severe combined immunodeficient (SCID) mice with HIV-1 encephalitis (HIVE). In HIVE mice, siRNA control (without target sequence, sicon) pre-transfected NPCs injected with HIV-1-infected MDM showed more astrocytic differentiation and less neuronal differentiation of NPCs as compared to NPC injection alone. siSTAT3 abrogated HIV-1-infected MDM-induced astrogliogenesis of injected NPCs. Collectively, these observations demonstrate that HIV-1-infected/activated MDM induces NPC astrogliogenesis through the STAT3 pathway. This study generates important data elucidating the role of brain inflammation in neurogenesis and may provide insight into new therapeutic strategies for HAD.

## Introduction

Active neurogenesis occurs throughout life and relies upon the proliferation, migration, and proper differentiation of neural stem/progenitor cells (NPCs) [1], [2]. Diminished adult neurogenesis is considered a potential factor in the pathogenesis of neurodegenerative diseases, including HIV-1-associated dementia (HAD), multiple sclerosis, Parkinson's, and Alzheimer's diseases [3], [4], [5], [6], [7], [8], [9]. Several studies have suggested a close linkage between the inflammatory reaction of the injured brain and the neurogenesis process [5], [10], [11], [12], [13]. Given that neurogenesis is affected by inflammatory states, we sought to determine mechanistically how HIV-mediated inflammatory may influence neurogenesis.

During HAD, HIV-1-infected and immune-activated brain mononuclear phagocytes (MP; perivascular macrophages and microglia) release many immune competent factors including cytokines, viral proteins and neurotoxins. These factors are involved in neuronal injury, drive central nervous system (CNS) inflammation, and may alter normal neurogenesis [14], [15], [16], [17], [18], [19]. It has been shown that HAD patients have fewer adult NPCs in the dentate gyrus of the hippocampus than non-infected subjects or HIV-1-infected patients without dementia [20], [21]. Moreover, analysis of the basal ganglia of severe combined immunodeficient (SCID) mice revealed inhibition of hippocampal neurogenesis following injection with HIV-1-infected human MP [7]. Our previous work demonstrated HIV-1-infected and immune-activated monocyte-derived macrophages (MDM) inhibit neurogenesis, while enhancing astrogliogenesis through secretion of inflammatory cytokines such as IL-1β and TNF-α [9]. However, the signaling pathways involved in this process are unknown.

The signal transducer and activator of transcription (STAT) 3 signaling pathway plays a critical role in NPC differentiation, particularly in enhancing astrocytic differentiation (astrogliogenesis) and inhibiting neuronal differentiation. Knockouts in the Janus Kinase-STAT3 (Jak-STAT3) pathway result in impaired astrocytic differentiation *in vivo* [22], [23], [24], [25]. In addition to the well-established role in development [24], [26], [27], the STAT3 pathway also contributes to microglial cell-induced astrogliogenesis [28], [29].

Using a primary human NPC culture system and a SCID HIV-1 encephalitis (HIVE) mouse model, we demonstrate that the STAT3 pathway is crucial for HIV-1-infected and immune-activated MDM-induced NPC astrogliogenesis and provide evidence that this effect is at least partially mediated by the action of TNF-α.

## Materials and Methods

### Monocyte cell culture and conditioned medium collection

Human monocytes were recovered from peripheral blood mononuclear cells (PBMCs) of HIV-1 and hepatitis B seronegative donors after leukopheresis and counter-current centrifugal elutriation [30]. Monocytes were cultured as adherent monolayer at a density of 1.1 ×10^6^ cells/well in 24-well plates and cultivated in Dulbecco's modified Eagles medium (DMEM, Invitrogen, Carlsbad, CA) with 10% heat-inactivated pooled human serum (Cambrex Bio Science, Walkersville, MD), 50 µg/ml gentamicin and/or 10 µg/ml ciprofloxacin (Sigma-Aldrich, St. Louis, IL) and 1000 U/ml highly purified recombinant human macrophage colony stimulating factor (MCSF, a generous gift from Wyeth Institute, Cambridge, MA).

Seven days after plating, human monocyte-derived macrophages (MDM) were infected with HIV-1 strain ADA at a multiplicity of infection (MOI) of 0.1virus/target cell [31]. Three to four days after infection HIV-1-infected and replicate uninfected MDM were then treated with/without lipopolysaccharide (LPS) (Sigma-Aldrich, 0.1 µg/ml) for 3 h. Cells were then rinsed two times with fresh DMEM to remove residual LPS, and serum-free DMEM was placed onto the MDM for 24 h. The MDM conditioned medium (MCM) was harvested, cleared of free-floating cells by centrifugation for 5 min at 1200 rpm, and stored at −80°C. MDM were fixed in 4% paraformaldehyde for p24 staining as described in the Immunocytochemistry section.

### Neural progenitor cell culture

Human cortical NPCs were isolated from human fetal brain tissue as previously described [32]. NPCs were cultured in substrate-free tissue culture flasks and grown as spheres in neurosphere initiation medium (NPIM), which consisted of X-Vivo 15 (BioWhittaker, Walkersville, ME) with N2 supplement (Invitrogen), neural cell survival factor-1 (NSF-1, BioWhittaker), basic fibroblast growth factor (bFGF, 20 ng/ml, Sigma-Aldrich), epidermal growth factor (EGF, 20 ng/ml, Sigma-Aldrich), leukemia inhibitory factor (LIF, 10 ng/ml, Chemicon, Temecula, CA), and 60 ng/ml N-acetylcysteine (Sigma-Aldrich). Cells were passaged at two-week intervals as previously described [32].

### Human neural progenitor cell differentiation

Following a protocol frequently used to induce neuronal differentiation of NPC [32], [33], [34], dissociated NPCs were plated on poly-D-lysine-coated cell culture dishes or coverslips (Sigma-Aldrich). Cells were cultured in NPIM for 24 h and subsequently changed to serum-free Neurobasal medium (Invitrogen) supplemented with B27 (Invitrogen, NB27) with or without MCM or cytokines. For the inhibition of TNF-α, MCM was pre-incubated with TNF-α soluble receptors R1 and R2 (TNF-R1R2, each 100 ng/ml, R&D Systems, Minneapolis, MN) for 1 h at 37°C. For siRNA transfection, pre-designed siRNA duplexes targeted against STAT3 mRNA (siSTAT3) were synthesized by Ambion Inc. (Austin, Texas). NPCs were transfected with 100 nM nonspecific control siRNA (sicon) or siSTAT3 in the presence of siImporter (Upstate Cell Signaling Solutions, Charlot-tesville, VI) according to the manufacturer's instructions. Twenty-four hours later, cells were treated with MCM to one to six days. Cells were fixed for immunocytochemical staining and protein or RNA was collected for Western blot or RT-PCR.

### Immunocytochemistry

Cells were fixed in 1∶1 methanol/acetone and washed in PBS as previously described [32]. Cells were then incubated overnight with mouse or rabbit anti-β-III-tubulin (Sigma-Aldrich, 1∶400) for the identification of neurons or rabbit anti-GFAP (glial fibrillary acidic protein, Dako, Carpinteria CA, 1∶1000) for the identification of astrocytes, followed by Alexa Fluor secondary antibodies, goat anti-mouse IgG Alexa Fluor 488 and goat anti-rabbit IgG Alexa Fluor 594 (Molecular Probes, Eugene, OR, 1∶400) for 1 h at room temperature. To detect HIV-1 infection, MDM were fixed in 4% paraformaldehyde for 20 min at room temperature and washed in PBS three times. Cells were then incubated overnight with p24 antibody (Dako, 1∶100), followed by goat anti-mouse IgG Alexa Fluor 488. All antibodies were diluted in PBS with 0.1% Triton X-100 and 2% BSA. Cells were counterstained with Hoechst 33342 (Sigma-Aldrich). Morphological changes were visualized and captured with a Nikon Eclipse E800 microscope equipped with a digital imaging system. Images were imported into Image-ProPlus, version 7.0 (Media Cybernetics, Sliver Spring, MD) for quantification. Ten to fifteen random fields (total 500–1000 cells per culture) of immunostained cells were manually counted using a 20× objective.

### Western blotting

Cells were rinsed twice with PBS and lysed by M-PER Protein Extraction Buffer (Pierce, Rockford, IL) containing 1× protease inhibitor cocktail (Roche Diagnostics, Indianapolis, IN). Protein concentration was determined using a BCA Protein Assay Kit (Pierce). Proteins (20–30 µg) were separated on a 10% SDS-polyacrylamide gel electrophoresis (PAGE) and then transferred to an Immuno-Blot polyvinylidene fluoride (PVDF) membrane (Bio-Rad, Hercules, CA). After blocking in PBS/Tween (0.01%) with 5% nonfat milk, the membrane was incubated with primary antibodies (phospho- and total-Jak1, Millipore; phospho- and total-STAT3, Cell Signaling Technologies; β-actin, GFAP, and β-III-tubulin, Sigma-Aldrich) overnight at 4°C followed by horseradish peroxidase-conjugated secondary antibodies (1∶10,000; Cell Signaling Technologies) and then developed using Enhanced Chemiluminescent (ECL) solution (Pierce). For data quantification the films were scanned with a CanonScan 9950F scanner and the acquired images were then analyzed on a Macintosh computer using the public domain NIH image program (developed at the U.S. National Institutes of Health and available on the internet at http://rsb.info.nih.gov/nih-image/).

### ELISA

Supernatants from MDM were collected for cytokine determination by an in house ELISA (for paired antibodies, R&D Systems). In Brief, 96-well microplates (Costar) were coated overnight at room temperature with a capture antibody in PBS. After three washes with PBS containing 0.05% Tween 20 (PBST), non-specific binding was blocked for 2 h with 1% BSA in PBS. Triplicate samples (100 µl) or a serial dilution of standards of human recombinant cytokine (R&D Systems) were applied to the wells and incubated overnight at 4°C. Plates were then incubated for 1 h at room temperature with the biotinylated detection antibody, followed by 30 min incubation with Streptavidin-HRP (R&D Systems). After three washes with PBST, 100 µl Substrate Solution (Tetramethylbenzidine, R&D Systems) was added to each well for 5 to 20 min, followed by the addition of 50 µl of Stop Solution (2N H_2_SO_4_, R&D System) to each well. The optical density of each well was determined using a microplate reader (Bio-Rad) set to 450 nm.

### NPC and MDM injections into SCID mice

Four-week-old male CB-17(SCID) mice were purchased from Jackson Laboratory (Bar Harbor, ME). Animals were maintained in sterile microisolator cages under pathogen-free conditions in the DRCI Animal Facility at UNMC, in accordance with ethical guidelines for care and use of laboratory animals set forth by the National Institutes of Health. NPCs were transfected with sicon or siSTAT3. Twenty-four hours later, NPCs were labeled with Qtracker (Invitrogen) following the manufacture's protocol. NPCs (sicon-NPC and siSTAT3-NPC) with or without HIV-1_ADA_-infected MDM (sicon-NPC+MDM and siSTAT3-NPC+MDM, 1∶4, total 5×10^5^ cells in 5 µl) were injected intracranially by stereotactic methods [35]. Four animals were included in each group. Seven days after injection, mice were euthanized with isoflurane and perfused transcardially with 25 ml of PBS and then 4% paraformaldehyde as previously described [36]. The brains were rapidly removed and immersed in freshly depolymerized 4% paraformaldehyde for 48 h and then cryoprotected by successive 24-h immersions in 10, 20, and 30% sucrose in Sorenson's phosphate buffer immediately before sectioning.

### Immunohistochemistry and image analysis

Fixed, cryoprotected brains were frozen and sectioned in the horizontal plane at 30 µm using a Cryostat (Leica Microsystems Inc., Bannockburn, IL), with sections collected serially in PBS. Antibodies to GFAP or β-III-tubulin were used for the detection of astrocytes or neurons. Double-immunofluorescence staining was performed using goat anti-mouse IgG Alexa Fluor 488 or goat anti-rabbit IgG 594 as a secondary antibody (Molecular Probes). All obtained images were imported into Image-ProPlus, version 7.0 (Media Cybernetics, Sliver Spring, MD) for quantifying levels of GFAP- and β-III-tubulin-positive staining. Four sections from each injection site were analyzed.

### Statistical analyses

Data were expressed as means ± SD (standard deviation). The data were evaluated statistically by analysis of variance (ANOVA) followed by the Tukey*-*test for paired observations. Significance was considered to be *p*≤0.05. To account for any donor-specific differences, all experiments were performed with NPCs and MDM from at least three donors. All assays were performed at least two times, with triplicate or quadruplicate samples in each assay.

## Results

### HIV-1-infected and/or LPS-activated MCM activate the Jak-STAT3 pathway

The STAT3 pathway plays an important role in NPC differentiation, particularly in enhancing astrocytic differentiation and inhibiting neuronal differentiation. Our previous work demonstrates HIV-1-infected and immune-activated macrophages inhibit NPC neurogenesis while enhancing astrogliogenesis *in vitro* and *in vivo* through secretion of inflammatory cytokines [9]. In this study we have further investigated if astrogliogenesis induced by secretion factors from HIV-1-infected and immune-activated MDM is through the STAT3 pathway. We used HIV-1-infected and/or LPS-activated MDM and human fetal cortical NPCs to test the effect of secreted factors from MDM on NPC differentiation.

Human fetal cortical NPCs were expanded as neurosphere in NPIM as previous described [32]. Under these conditions, over 90% of NPCs were immunopositive for the cytoskeletric protein nestin, a marker for multipotential precursors (Figure S1A–C) whereas less than 10% of the cells expressed the neuronal marker β-III-tubulin or the astrocytic marker GFAP (data not shown). This suggests that the majority of the cells were in an undifferentiated state.

**Figure 1:**
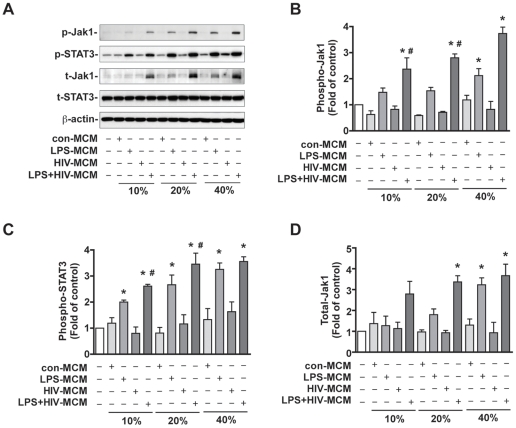
LPS-activated and/or HIV-1-infected MCM induce dose-dependent Jak-STAT3 activation in Human NPCs. **A.** Human NPCs were treated with 10, 20, and 40% con-MCM, LPS-MCM, HIV-MCM, or LPS+HIV-MCM for 24 h. Expression of phospho-Jak1 (p-Jak1), phospho-STAT3 (p-STAT3), total-Jak1 and total-STAT3 were detected by Western blotting. β-actin was used as a loading control. Results are representative of three independent experiments. **B–D.** The films were scanned and the acquired images were analyzed using the public domain NIH image program for data quantification. Expression of phospho-Jak1 (B), phospho-STAT3 (C), and total-Jak1 (D) were normalized to β-actin. Data is presented as fold of control expression. Results are average of three independent donors. * p<0.05 in comparison to control, # p<0.05 in comparison to LPS-MCM of the same concentration.

To determine whether MCM activate the Jak-STAT3 pathway in NPCs, immunoblots were performed on cell extracts treated with or without con-MCM, LPS-MCM, HIV-MCM, and LPS+HIV-MCM. NPCs were cultured in NPIM for 24 hours and subsequently changed to NB27 medium with different concentrations of MCM (10, 20, and 40%) for an additional 24 hours (Figure 1A). The quantification results show both LPS-MCM and LPS+HIV-MCM induced a dose-dependent increase of Jak-1 (Figure 1B) and STAT3 (Figure 1C) activation. Although HIV-MCM did not induce phosphorylation of Jak1 and STAT3, LPS+HIV-MCM more robustly activated Jak1 and STAT3 as compared to LPS-MCM (Figure 1B–C), suggesting HIV infection potentiates LPS-MCM-induced Jak/STAT3 activation. In addition, both LPS-MCM and LPS+HIV-MCM induced increases of total-Jak1 expression as compared to control (Figure 1D). The MCM effect on total-Jak1 was similar to that on Jak1 phosphorylation (40% LPS+HIV-MCM induced 3.5-fold increase in phospho-Jak1 and 3.6-fold increase in total-Jak1), suggesting that the increase in MCM-induced Jak1 phosphorylation may be due to the overall increase of total-Jak1.

**Figure 2:**
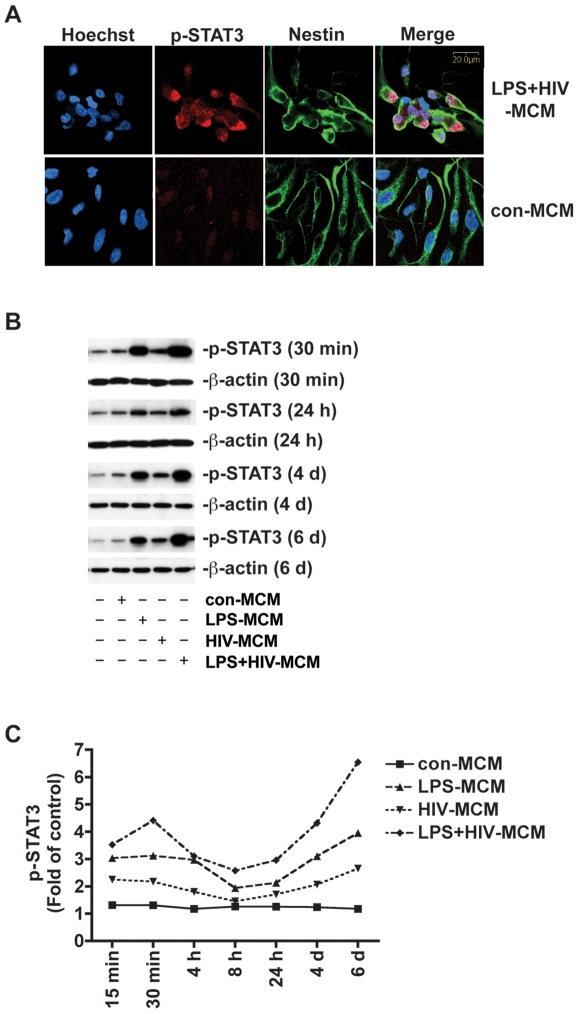
LPS-activated and/or HIV-1-infected MCM induce sustained STAT3 activation in human NPCs. **A.** Human NPCs were treated with or without 20% LPS+HIV-MCM for 24 h. Cells were immunolabeled with antibodies to NPC marker Nestin (green) and phospho-STAT3 (p-STAT3, red). Nuclei were stained with Hoechst (blue). Results are representative of two independent experiments. Original magnification is ×60. **B.** NPCs were treated with 20% con-MCM, LPS-MCM, HIV-MCM, or LPS+HIV-MCM for 15 min, 30 min, 4 h, 8 h, 24 h, 4 d, and 6 d. Expression of p-STAT3 was detected by Western blotting and normalized to β-actin. **C.** The films were scanned and the acquired images were analyzed using the public domain NIH image program for data quantification. Expression of p-STAT3 was normalized to β-actin. Data is presented as fold of control expression. Results are representative of two independent experiments.

To confirm the effect of MCM is time-dependent, we treated NPCs with 20% control-MCM, LPS-MCM, HIV-MCM, and LPS+HIV-MCM at time points ranging from 15 minutes to six days and immunoblots were performed on cell extracts (Figure 2B). The quantification results show that MCM-induced STAT3 activation began at 15 minutes post treatment and first peaked between 15–30 minutes (Figure 2C). Activation of STAT3 was sustained at all time points indicated up until six days and increased during cell differentiation. Maximal activation of STAT3 was observed at six days post treatment. (Figure 2C).

To confirm that LPS+HIV-MCM-induced STAT3 activation occurs in NPCs, we performed immunocytochemical studies with NPC culture using a phospho-STAT3 antibody and an antibody against nestin, a neural progenitor cell marker. Upon LPS+HIV-MCM treatment, we found obvious nuclear-localized phosphorylation of STAT3 as compared to con-MCM group (Figure 2A). The results also show p-STAT3 highly co-localized with nestin-positive cells (Figure 2A and Figure S1D–F). To detect if LPS+HIV-MCM induce STAT3 activation in differentiated astrocytes, which exist in NPC culture at a low percentage (less than 10%), we treated NPCs with LPS+HIV-MCM and did immunocytochemical studies using antibodies for p-STAT3 and GFAP, an astrocyte marker (Figure S1G–I). The results show that although some GFAP-positive cells were also p-STAT3-positive, the majority of p-STAT3-positive cells were GFAP-negative (Figure S1I). So we consider that the p-STAT3 signal observed in Western blot analysis originated primarily from nestin-positive NPCs.

### HIV-1-infected and/or LPS-activated MCM-induced astrogliogenesis is through the Jak-STAT3 pathway

**Figure 3:**
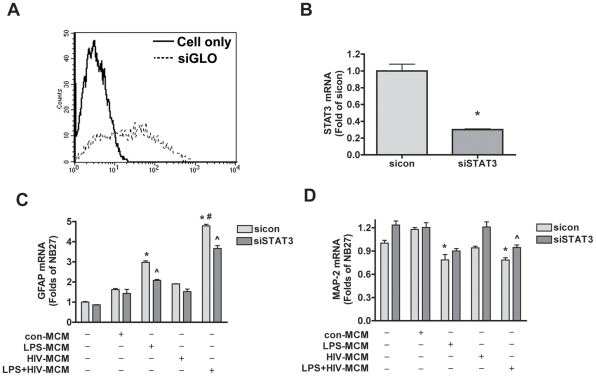
siRNA for STAT3 inhibits LPS-activated and/or HIV-1-infected MCM-induced astrogliogenesis by realtime RT-PCR. **A.** NPCs were transfected with fluorescence-labeled control siRNA (siGLO) and analyzed by flow cytometry. The transfection efficiency of siGLO reached 60% at 24 h post-transfection. **B.** NPCs were transfected with siSTAT3 or sicon for 24 h. STAT3 mRNA expression were detected by TaqMan realtime RT-PCR and normalized to GAPDH as an internal gene expression control. Data are presented as fold of sicon. * p<0.05 in comparison to sicon. **C**–**D.** NPCs were transfected with siSTAT3 or sicon for 24 h and then differentiated in NB27 medium with or without 20% con-MCM, HIV-MCM, LPS-MCM, or LPS+HIV-MCM for 6 d. GFAP (C) and MAP-2 (D) mRNA expression were detected by TaqMan realtime RT-PCR and normalized to GAPDH as an internal gene expression control. Data are presented as fold of NB27. * p<0.05 in comparison to NB27, # p<0.05 in comparison to LPS-MCM, ∧ p<0.05 in comparison to sicon of the same condition.

Next we investigated the role of the STAT3 pathway in MCM-mediated astrocytic differentiation by inhibition of STAT3 expression using siRNA. To evaluate the transfection efficiency in human NPCs, cells were first transfected with fluorescence-labeled control siRNA (siGLO) and analyzed by flow cytometry (Figure 3A). The transfection efficiency of siGLO reached 60% 24 hours post-transfection (Figure 3A). Gene expression analysis using realtime RT-PCR found that siSTAT3 decreased 70% of the STAT3 mRNA expression as compared to sicon-transfected NPCs at 24 h post-transfection (Figure 3B). siSTAT3 showed the similar effect on STAT3 mRNA expression at 48 h post-transfection (data not shown), so we started MCM treatment at 24 h post-transfection for the following experiments.

To test whether HIV-1-infected and/or activated MDM-induced astrogliogenesis is through the STAT3 pathway, NPCs were transfected with siSTAT3 or sicon and then differentiated with or without HIV-1-infected and/or LPS-activated MCM for 6 days. The effect of siSTAT3 on astrocytic differentiation was first determined by realtime RT-PCR (Figure 3C, D). NPCs treated with LPS-MCM and LPS+HIV-MCM displayed increased GFAP (astrocyte marker) mRNA expression (Figure 3C) and decreased MAP-2 (neuronal marker) mRNA expression (Figure 3D), demonstrating an induction of astrogliogenesis and inhibition of neurogenesis. Although HIV-MCM did not induce a significant effect on GFAP mRNA expression, LPS+HIV-MCM displayed a more dramatic increase of GFAP mRNA expression as compared to LPS-MCM (Figure 3C). Conversely, knockdown of STAT3 by siRNA inhibited the LPS-MCM and LPS+HIV-MCM-induced increase of GFAP expression (Figure 3C) and the decrease of MAP-2 expression (Figure 3D) at six days post-transfection, suggesting that decreased STAT3 expression abrogates LPS-MCM and LPS+HIV-MCM-induced astrogliogenesis.

**Figure 4 pone-0019439-g004:**
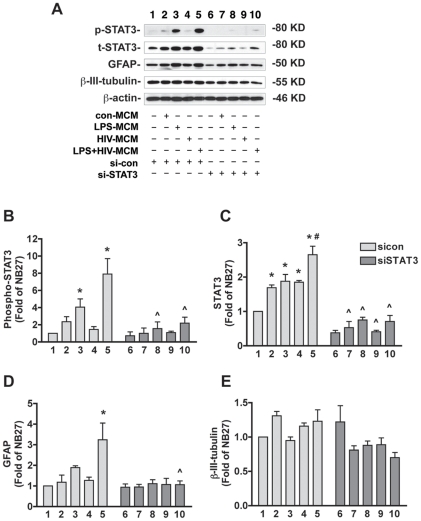
siRNA for STAT3 inhibits LPS-activated and/or HIV-1-infected MCM-induced STAT3 activation and astrogliogenesis. **A.** NPCs were transfected with siSTAT3 or sicon for 24 h and then differentiated in NB27 medium with or without 20% con-MCM, HIV-MCM, LPS-MCM, or LPS+HIV-MCM for 6 d. Expression of p-STAT3, t-STAT3, GFAP, and β-III-tubulin was detected by Western blotting. Results are representative of three independent experiments. **B**–**E.** The films were scanned and the acquired images were analyzed using the public domain NIH image program for data quantification. Expression of phospho-STAT3 (B), STAT3 (C), GFAP (D), and β-III-tubulin (E) were normalized to β-actin. Data is presented as fold of NB27 expression. Results are average of three independent donors. * p<0.05 in comparison to NB27, # p<0.05 in comparison to LPS-MCM, ∧ p<0.05 in comparison to sicon of the same condition.

We next utilized Western blot to examine whether siSTAT3 could modulate HIV-1-infected and/or LPS-activated MCM-induced STAT3 activation and NPC differentiation (Figure 4). In agreement with mRNA expression, we found that STAT3 protein expression was decreased in siSTAT3-transfected NPCs (Figure 4A, C). While LPS-MCM and LPS+HIV-MCM significantly increased STAT3 activation, siSTAT3 dramatically decreased LPS-MCM and LPS+HIV-MCM-induced STAT3 phosphorylation (Figure 4A, B). Furthermore, LPS-MCM and LPS+HIV-MCM increased GFAP expression, while siSTAT3 inhibited these changes (Figure 4A, D). However, we did not observe a decrease of β-III-tubulin (neuronal marker) expression by LPS-MCM and LPS+HIV-MCM stimulation. The possible explanation is that Western blotting may not be sensitive enough to show the changes of β-III-tubulin protein.

**Figure 5:**
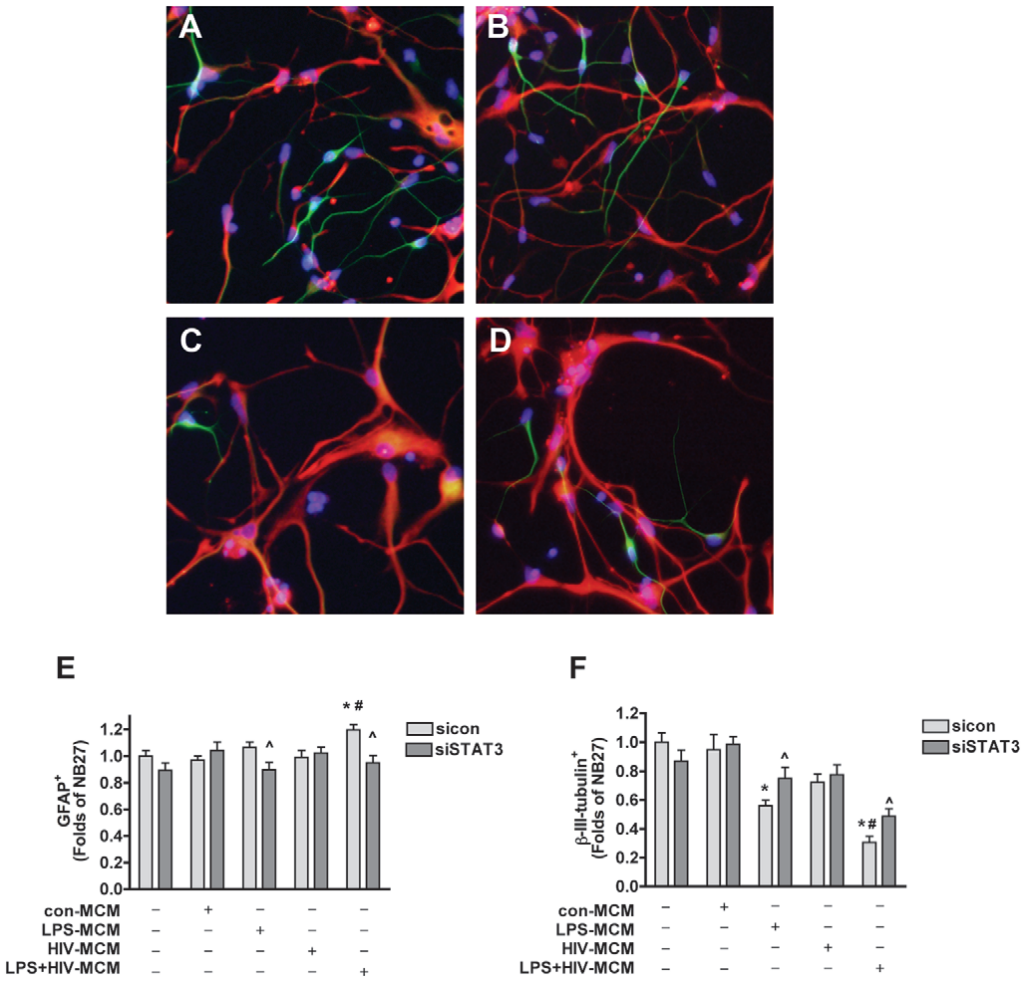
siRNA for STAT3 inhibits LPS-activated and/or HIV-1-infected MCM-induced increase of astrocyte and decrease of neuronal proportions. NPCs were transfected with siSTAT3 or sicon for 24 h and were then differentiated in NB27 medium with or without 20% con-MCM, HIV-MCM, LPS-MCM, or LPS+HIV-MCM for 6 d. A–D. Representative fluorescence overlay micrographs display the morphology of neurons (green) and astrocytes (red) in sicon-transfected NPCs without treatment (sicon-NB27, A), siSTAT3-transfected NPCs without treatment (siSTAT3-NB27, B), sicon transfected NPCs treated with LPS+HIV-MCM (C) or siSTAT3-transfected NPCs treated with LPS+HIV-MCM (D). Nuclei were stained with Hoechst (blue). E–F. GFAP (E) or β-III-tubulin (F) positive cells were quantified; data is presented as fold of sicon-NB27 expression. Results are representative of two independent experiments. * p<0.05 in comparison to sicon-NB27, # p<0.05 in comparison to LPS-MCM, ∧ p<0.05 in comparison to sicon of the same condition.

To validate whether the increase of GFAP is due to the increase in the number of astrocytes, the effect of the STAT3 pathway on MCM-induced astrogliogenesis was further tested by immunocytochemistry. NPCs were transfected with siSTAT3 and sicon and then differentiated with or without HIV-1-infected and/or immuno-activated MCM for six days. Expression of differentiation markers was evaluated by immunostaining with antibodies against β-III-tubulin or GFAP (Figure 5). LPS+HIV-MCM treatment significantly increased the proportion of the GFAP-positive cells (Figure 5E), while LPS- and LPS+HIV-MCM treatment significantly decreased the proportion of the β-III-tubulin-positive cells (Figure 5F). As expected, siSTAT3 significantly inhibited LPS+HIV-MCM-induced astrogliogenesis (Figure 5E, from 1.20 ± 0.13 to 0.95 ± 0.19, *p* < 0.01) and partially abrogated the inhibition of neurogenesis induced by LPS+HIV-MCM (Figure 5F, from 0.31 ± 0.13 to 0.49 ± 0.18, *p* < 0.01).

Taken together, these results suggest that HIV-1-infected and immune-activated MCM promote astrogliogenesis through the STAT3 pathway.

### TNF-α contributes to immune-activated and/or HIV-1-infected MCM-induced STAT3 activation and astrogliogenesis

**Figure 6:**
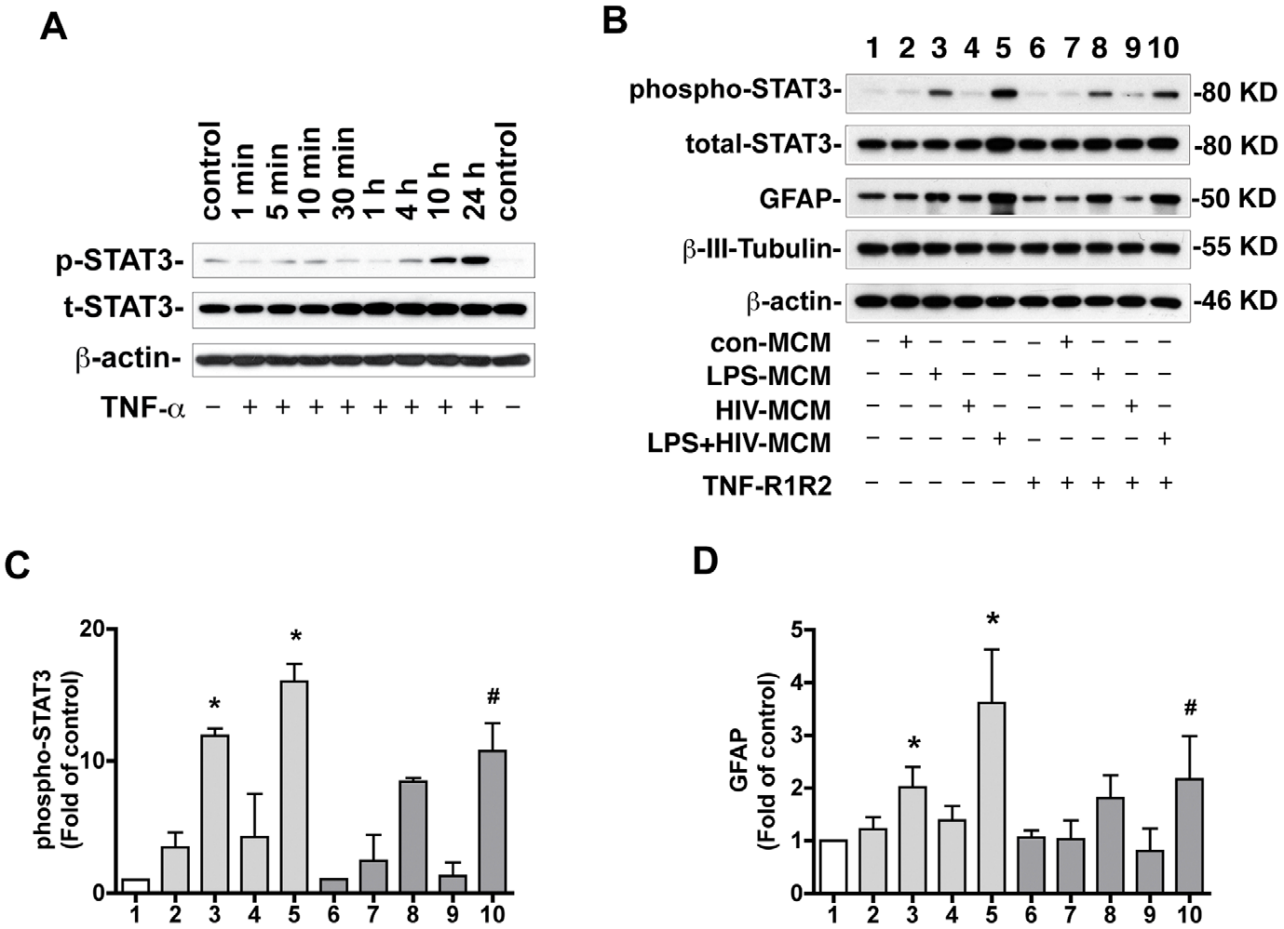
LPS-activated and/or HIV-1-infected MCM-induced STAT3 activation and astrogliogenesis are partially through TNF-α. **A.** NPCs were treated with TNF-α (20 ng/ml) for 1 min, 5 min, 10 min, 30 min, 4 h, 10 h, and 24 h. Expression of p-STAT3 and t-STAT3 was detected by Western blotting. β-actin was used as a loading control. Results are representative of three independent experiments. **B**. NPCs were differentiated in NB27 with 20% MCM with or without incubation with TNF-R1R2 (100 ng/ml) for 6 d. Expression of total-STAT3, phospho-STAT3, GFAP, and β-III-tubulin was detected by Western blotting. Results are representative of three independent experiments. **C**–**D**. The films were scanned and the acquired images were analyzed using the public domain NIH image program for data quantification. Expression of phospho-STAT3 (B) and GFAP (C) were normalized to β-actin. Data is presented as fold of NB27 expression. Results are average of three independent donors. * p<0.05 in comparison to NB27, # p<0.05 in comparison to no TNF-R1R2-treatment of the same condition.

Previous studies demonstrated that TNF-α is elevated in immune activated and/or HIV-1-infected MDM and contributes to LPS-activated and/or HIV-infected MCM-induced astrogliogenesis [9]. Thus we tested whether TNF-α could induce STAT3 activation. We treated NPCs with TNF-α (20 ng/ml) at time points ranging from 1 min to 24 h. The results show that TNF-α induced late activation of STAT3 (started at 4 h and sustained to 24 h, Figure 6A).

To assess whether TNF-α is responsible for inducing STAT3 activation in NPCs treated with HIV-1-infected and/or LPS-activated MCM, MCM were pre-incubated with TNF soluble receptors (100 ng/ml) before the treatment. The results show that TNF-R1R2 partially reduced LPS+HIV-MCM-induced STAT3 activation (from 8.26±1.00 to 6.03±1.05 fold as compared to NB27, Figure 6B–C). Pre-incubation with TNF-R1R2 also partially reduced LPS+HIV-MCM-induced astrocytic differentiation as shown by GFAP expression (from 4.22±0.52 to 2.74±0.47 fold as compared to NB27, Figure 6B, D). These results indicate that TNF-α, derived from immune-activated and/or HIV-1-infected MDM, contributes to MCM-induced astrogliogenesis via the STAT3 pathway.

### HIV-1-infected MDM morphology and analysis of cytokine levels in LPS-activated and/or HIV-1-infected MCM

To evaluate HIV infection efficiency at the time of MCM collection, we utilized immunostaining for the p24 protein of HIV, the capsid protein of the virus.

Seven days after plating, we exposed MDM to HIV-1 strain ADA at a multiplicity of infection (MOI) of 0.1virus/target cell [31]. Three to four days after exposure to HIV-1, HIV-1-infected MDM merged into multi-nuclear giant cells (Figure S2D-F). These cells were then stimulated with LPS for 3 h and MCM was harvested 24 h after stimulation. The HIV-1 infection efficiency was assessed by immunostaining for the p24 protein of HIV-1. An average of 61.1±9.6% of the cells were positive for the expression of HIV-1 p24 in the HIV-1-infected group (HIV, Figure S2D-F). The LPS-stimulated group showed a lower trend of HIV-1 infection efficiency (48.1±20.5%, HIV+LPS, Figure S2G–H), but not statistically different when compared to the HIV-1-infected group without LPS stimulation (Figure S2J).

TNF-R1R2 only partially reduced LPS+HIV-MCM-induced STAT3 activation and astrogliogenesis, so we used ELISA to investigate several inflammatory factors secreted by macrophages/microglia that are considered to induce astrogliogenesis. Among the cytokines examined, the protein level of LIF in MCM was very low (less than 3 pg/ml, data not shown). IL-6, IL-1β, and TNF-α showed the similar patterns of expression: LPS induced IL-6, IL-1β, and TNF-α production by MDM (0.734±0.710 ng/ml for IL-6, 0.026±0.007 ng/ml for IL-1β, 0.659±0.098 ng/ml for TNF-α), LPS+HIV induced a more dramatic increase of cytokine production (2.396±1.081 ng/ml for IL-6, 0.212±0.104 ng/ml for IL-1β, 4.180±3.043 ng/ml for TNF-α, Figure S2K–M). These observations suggest that although HIV-1 infection alone did not induce a significant effect on IL-6, IL-1β, and TNF-α expression, HIV-1 infection potentiated LPS-stimulated cytokine production. The observed cytokine expression pattern correlates with LPS-MCM and LPS+HIV-MCM-induced STAT3 activation and astrogliogenensis, suggesting IL-6 and IL-1β may also contribute to MCM-induced STAT3 activation and astrogliogenesis.

### siSTAT3 inhibits HIV-1-infected MDM-induced NPC astrogliogenesis in HIVE mice

**Figure 7:**
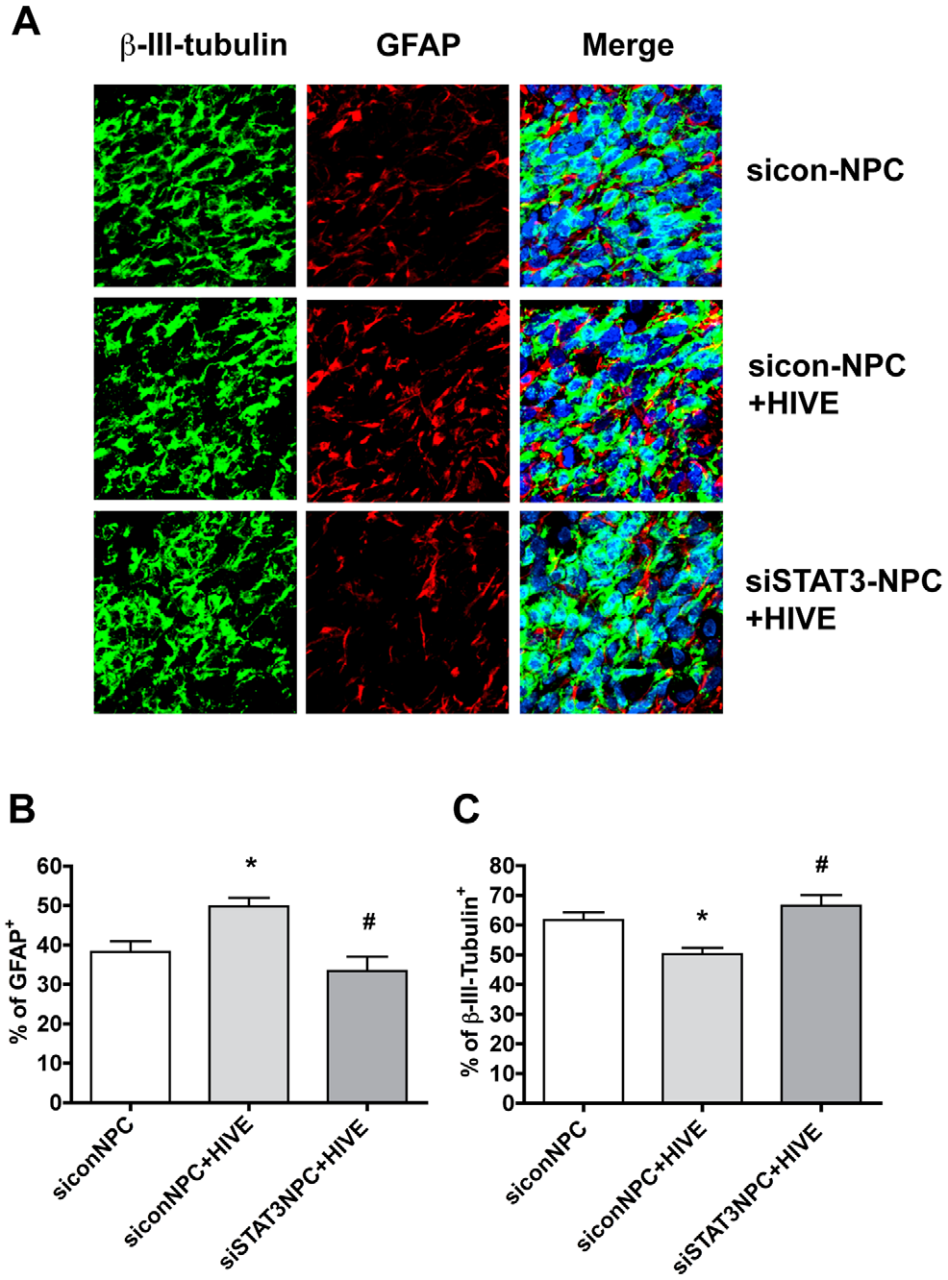
siSTAT3 inhibits HIV-1-infected MDM-induced NPC astrogliogenesis. NPCs were transfected with siSTAT3 or sicon and labeled with Qtracker. Cells were then intracranially injected into the basal ganglia of SCID mice with or without HIV-1_ADA_-infected MDM. Severn days after injection, NPC differentiation was identified using immunostaining in serial 30 µm brain sections. **A.** Serial brain sections from mice injected with sicon-NPC, sicon-NPC injected with HIV-1-infected MDM (sicon-NPC+HIVE), and siSTAT3-NPC+HIV-1-infected MDM (siSTAT3-NPC+HIVE) were immunolabeled with antibodies to GFAP (red) and β-III-tubulin (green). Original magnification is 60 ×. **B-C.** Neuronal or astrocytic differentiation was quantified by determining the percentage of GFAP-positive or β-III-tubulin-positive cells. Data is presented as the mean ± SEM and shown as the percentage of GFAP-positive cells (**B**), and the percentage of β-III-tubulin-positive cells (**C**) in the injection area. * p<0.05 in comparison to sicon-NPC. # p<0.05 in comparison to sicon-NPC+HIV-MDM.

To investigate the role of the STAT3 pathway in HIV-1-infected macrophage-mediated NPC astrogliogenesis *in vivo*, we used an HIVE SCID mouse model [9]. Human NPCs transfected with siSTAT3 or sicon were intracranially injected into the basal ganglia of SCID mice with or without HIV-1_ADA_-infected MDM. Seven days after injection, NPC differentiation was identified throughout the injected hemisphere by immunostaining in serial 30 µm brain sections (Figure 7). Confocal images show that the injected human NPCs survived and differentiated into neurons (β-III-tubulin-positive, green, Figure 7A) and astrocytes (GFAP-positive, red, Figure 7A). Neuronal and astrocytic differentiation were quantified by determining the percentage of GFAP-positive or β-III-tubulin-positive cells in the injection area. HIV-1_ADA_-infected MDM increased astrocytic differentiation (Figure 7B) and decreased neuronal differentiation (Figure 7C) compared to NPC injected alone. Additionally, siSTAT3 abrogated HIV-1-infected MDM induced-astrocytic differentiation of NPCs as compared to NPCs transfected with sicon. This data further confirms that HIV-1-infected MDM induce NPC astrocytic differentiation *in vivo* via activation of the STAT3 pathway, while decreased action of the STAT3 pathway lowers astrogliogenesis and induces more neuronal differentiation of NPCs.

## Discussion

Previous work in our lab has demonstrated that HIV-1-infected and immune-activated MDM inhibit NPC neurogenesis, while enhancing astrogliogenesis both *in vitro* and *in vivo*. To extend on our previous findings, we examined the function of the Jak-STAT3 pathway, a crucial part of the astrogliogenic machinery, in HIV-1-infected and/or immune-activated MCM-induced NPC differentiation. We found that LPS-MCM induces activation of STAT3 and LPS+HIV-MCM induces more dramatic activation of STAT3 as compared to LPS-MCM (Figure 1–2). siRNA-mediated knockdown of STAT3 expression results in a reduction of MCM-induced STAT3 activation and astrocytic differentiation (Figure 3–5) *in vitro*. siSTAT3 also reduced HIV-1-infected MDM-induced astrogliogenesis *in vivo* (Figure 7). Furthermore, inflammatory cytokines (including IL-6, IL-1β and TNF-α) produced by LPS-activated and/or HIV-1-infected MDM may contribute to MCM-induced STAT3 activation and astrocytic differentiation (Figure 6 and Figure S2). Thus, we report here that HIV-1-infected and immune-activated MDM promote astrogliogenesis through the secretion of cytokines via the Jak-STAT3 pathway.

Activation of the Jak-STAT3 signaling pathway is considered one of the main mechanisms promoting astrocytic differentiation and inhibiting neuronal differentiation of neural stem/progenitor cells [25], [26], [37], [38]. Cytokines activate members of the Jak family, which in turn activate, by tyrosine phosphorylation, one or more members of the STAT family of transcription factors. Dimerized STAT3 translocates to the nucleus and binds to the GFAP promoter activating the transcription of GFAP [26], [37]. Previous work from other laboratories has demonstrated microglial-derived soluble factors induce astrocytic differentiation through the STAT3 pathway [29]. We further investigated the potential role of the STAT3 pathway in HIV-infected and immune-activated MDM. In this study, we found that although HIV-1-infected MCM does not induce STAT3 activation and subsequent astrocytic differentiation, HIV infection potentiates LPS-activated MCM-induced STAT3 activation as compared to LPS-MCM (Figure 1-2). The activation of Jak/STAT3 is correlated with the up-regulation of GFAP transcription and protein expression, which indicates MCM-induced astrogliogenesis may be through the Jak1-STAT3 pathway. The requirement of this pathway in astrocytic differentiation was investigated by inhibition of STAT3 expression. siRNA targeting STAT3 down-regulated STAT3 expression (Figure 3–4), decreased STAT3 activation induced by MCM (Figure 4), and blocked astrocytic differentiation induced by LPS-MCM and LPS+HIV-MCM (Figure 3–5) *in vitro*. Furthermore, siSTAT3 reduced HIV-1-infected MDM induced astrogliogenesis in the SCID mouse HIVE model (Figure 7). In conclusion, our work further demonstrates that HIV-1-infected and immune-activated MDM induce astrogliogenesis through the Jak1-STAT3 pathway.

In mammals, the Jak-STAT3 pathway is the principal signaling mechanism for a wide array of cytokines and growth factors. Members of the IL-6 cytokine family such as, leukemia inhibitory factor (LIF), IL-6 and ciliary neurotrophic factor (CNTF), can activate the Jak-STAT signaling pathway and promote astroglial differentiation [26], [27]. Nakanishi et al. have reported that activated microglia promote astrocytic differentiation of NSCs through the release of cytokines, IL-6 and LIF [29]. We detected IL-6 and LIF expression by HIV-1-infected and/or LPS-activated MDM by real-time RT-PCR (data not shown) and ELISA. ELISA results show that although LIF mRNA is detected in MDM, the protein level of LIF in MCM is very low. Data demonstrate that the level of IL-6 is elevated in LPS-MCM and LPS+HIV-MCM (Figure S2K) and the expression pattern correlates with STAT3 activation and astrogliogenensis, suggesting IL-6 may contribute to MCM-induced STAT3 activation and astrogliogenesis.

Our previous studies showed that TNF-α is produced by HIV-1-infected and/or LPS-activated MDM and contributes to HIV-1-infected and/or LPS-activated MCM-induced astrogliogenesis [9]. In this study, we further demonstrated TNF-α induces STAT3 activation in NPCs (Figure 6A). TNF-R1 and R2 partially abrogate HIV-1-infected and/or LPS-activated MCM-induced STAT3 activation and astrogliogenesis (Figure 6B–D), suggesting TNF-α-derived from HIV-1-infected and/or LPS-activated-MDM may contribute to MCM-induced STAT3 activation and astrogliogenesis. However, TNF-α-induced STAT3 activation does not coincide with the LPS-MCM and LPS+HIV-MCM-induced STAT3 activation profile. While TNF-α-induced STAT3 activation starts four hours post-treatment (Figure 6A), LPS-MCM and LPS+HIV-MCM-induced STAT3 activation began at 15 minutes and was sustained until six days (Figure 2). One possible explanation for this temporal variance is that other soluble factors, such as IL-6 and LIF, released from MDM may contribute to MCM-induced early time point activation of STAT3. Preliminary data from our lab demonstrated that human recombinant IL-6 induces a moderate increase of STAT3 activation, while LIF induces a dramatic activation of STAT3 at early time point (15 to 30 min). However, the protein level of LIF in MCM is very low as measured by ELISA. The correlated expression pattern of IL-6 and STAT3 activation induced by LPS- and LPS+HIV-MCM suggests IL-6 may contribute to MCM-induced STAT3 activation at early time points. The role of IL-6 and LIF in MCM-induced STAT3 activation and astrogliogenesis may need to be further investigated.

Both TNF-α (Figure 6A) and IL-1β (data not shown) induce STAT3 activation at delayed time points, suggesting these cytokines play an indirect role. Unpublished data from our lab show that IL-1β and TNF-α induce NPC's production of LIF and IL-6, which could activate STAT3. These intermediate cytokines may contribute to the delayed and sustained activation of STAT3 and subsequent astrogliogenesis induced by TNF-α and IL-1β. However, the mechanisms by which IL-1β and TNF-α induce production of LIF and IL-6 and subsequent astrogliogenesis require further investigation.

The role played by microglia/macrophage in the regulation of neurogenesis under specific pathological conditions is a matter of hot debate [5], [10], [28], [29], [39], [40]. In HAD, MP are the principal cells infected by HIV and major mediators of the inflammatory response within the brain. Following HIV-1 infection and immune activation, MP undergo functional alterations that lead to the secretion of cytokines thus inducing astrogliogenesis [9]. We reasoned the interaction between MP and NPCs is a major aspect for how neurogenesis is affected in a diseased brain. Our data, consistent with findings from other groups [5], [12], [29], [40], suggest that acutely activated microglia/macrophage increase the percentage of GFAP-positive cells produced from NPCs via the activation of the Jak-STAT3 pathway. Importantly, we also demonstrated that once immune-activated, HIV-1-infected MP induce more GFAP-positive cells from NPCs. This may contribute to astrogliosis, an important pathological feature of HAD. Although the enhancement of astrocyte generation could be regarded as a potentially beneficial mechanism in neurodegenerative diseases given the role of astrocytes in synapse formation and stabilization, identification of suitable tools to direct NPCs toward the neuronal phenotype could represent a new strategy to enforce endogenous brain regenerative processes.

A previous study using NSC culture showed that suppression of STAT3 directly induces neurogenesis and inhibits astrogliogenesis [41]. In this study, over-expression of a dominant negative form of STAT3, STAT3F, resulted in significantly increased expression of proneural bHLH transcription factors, such as Math1, Ngn3, and NeuroD and decreased expression of Notch1-3, Hes1, and Hes5 within the Notch pathway for gliogenesis [41]. A recent study, using neural stem cell (NSC) isolated from STAT3^flox/flox^ mouse embryos, also revealed that elimination of STAT3 in NSC promoted neurogenesis and inhibited astrogliogenesis through the down-regulation of notch1, notch2 and hes5 [38]. In our study, we used siRNA-targeting STAT3 to down-regulate LPS-MCM and LPS+HIV-MCM-induced STAT3 activation and subsequent astrogliogensis. siSTAT3 also partially reversed LPS-MCM and LPS+HIV-MCM-induced inhibition of neurogenesis.

In conclusion, we have demonstrated that HIV-1-infected and/or immune activated MDM promote astrogliogenesis through the secretion of cytokines such as TNF-α and that their effects are mediated by activation of STAT3 signaling. These findings further suggest that brain inflammation disrupts neurogenesis in HAD. NPCs have been experimentally used to repair the damaged nervous system, either by transplantation of cells grown *in vitro* or by activation of endogenous NPCs. A complete understanding of how NPCs function under inflammatory conditions will allow us to modulate the environment and devise therapeutic strategies to enhance recovery from various CNS disorders. The interaction between activated macrophage and the STAT3 pathway raises the possibility that neuronal production in the brain can be regulated, at least in part, by manipulation of the STAT3 pathway. This pathway represents a potential drug target for promoting endogenous neurogenesis for the treatment of patients suffering from NeuroAIDS.

## Supporting Information

Figure S1 **Characterization of human cortical NPCs. A–C.** Human fetal cortical NPCs were expanded as neurosphere in NPIM. Cells were dissociated and plated on poly-D-lysine-coated cover slips for 24 h. Cells were fixed and stained for Nestin (red, A). Nuclei were stained using Hoechst 33342 (blue, B). C shows merge of A and B. Original magnification is 20 ×. Results are representative of two donors. **D–I.** NPCs were cultured in NPIM overnight and were then treated with 20% LPS+HIV MCM in NB27 for 24 h. Cells were immunolabeled with antibodies to phospho-STAT3 (p-STAT3, red, E, F, H and I) and Nestin (green, D and F) or astrocyte marker GFAP (green, G and I). Nuclei were stained with Hoechst (blue in merged pictures F and I). Results are representative of two independent experiments. Original magnification is 40 ×.
(TIF)

Figure S2 **LPS-activated and/or HIV-1-infected MDM induce cytokine production.** **A–I.** HIV-1 infection. MDM were infected with HIV-1_ADA_ for 3–4 days and then stimulated with LPS (100 ng/ml) for 3 h. Cells were stained with antibody to p24 (HIV-1 infection marker, green), conjugated with anti-mouse Alexa fluo 488 nm secondary antibody. Hoechst 33342 was used for nuclear staining. A–C show control uninfected MDM. D–F show HIV-1-infected MDM (HIV). G-I show LPS-activated and HIV-1-infected MDM (HIV+LPS). Panels are representative of three separate donors. Original magnification is 20 ×. **J.** HIV-1 infection was quantified by determining the percentage of p24-positive cells of seven to ten random microscopy fields. Data is presented as the mean ± SEM. **K–M.** HIV-infected and/or LPS-activated MCM were collected and measured for levels of IL-6 (K), IL-1β (L), and TNF-α (M) by ELISA. Data is presented as the mean ± SD. Results represent the average of four donors. * p<0.001 in comparison to con-MCM, # p<0.001 in comparison to LPS-MCM.
(TIF)
