# Supplementary figures and images for: HIV-1-Infected and Immune-Activated Macrophages Induce Astrocytic Differentiation of Human Cortical Neural Progenitor Cells via the STAT3 Pathway
Source: PLoS One. 2011 May 27;6(5):e19439. doi: 10.1371/journal.pone.0019439 (PMC3103496; doi:10.1371/journal.pone.0019439)

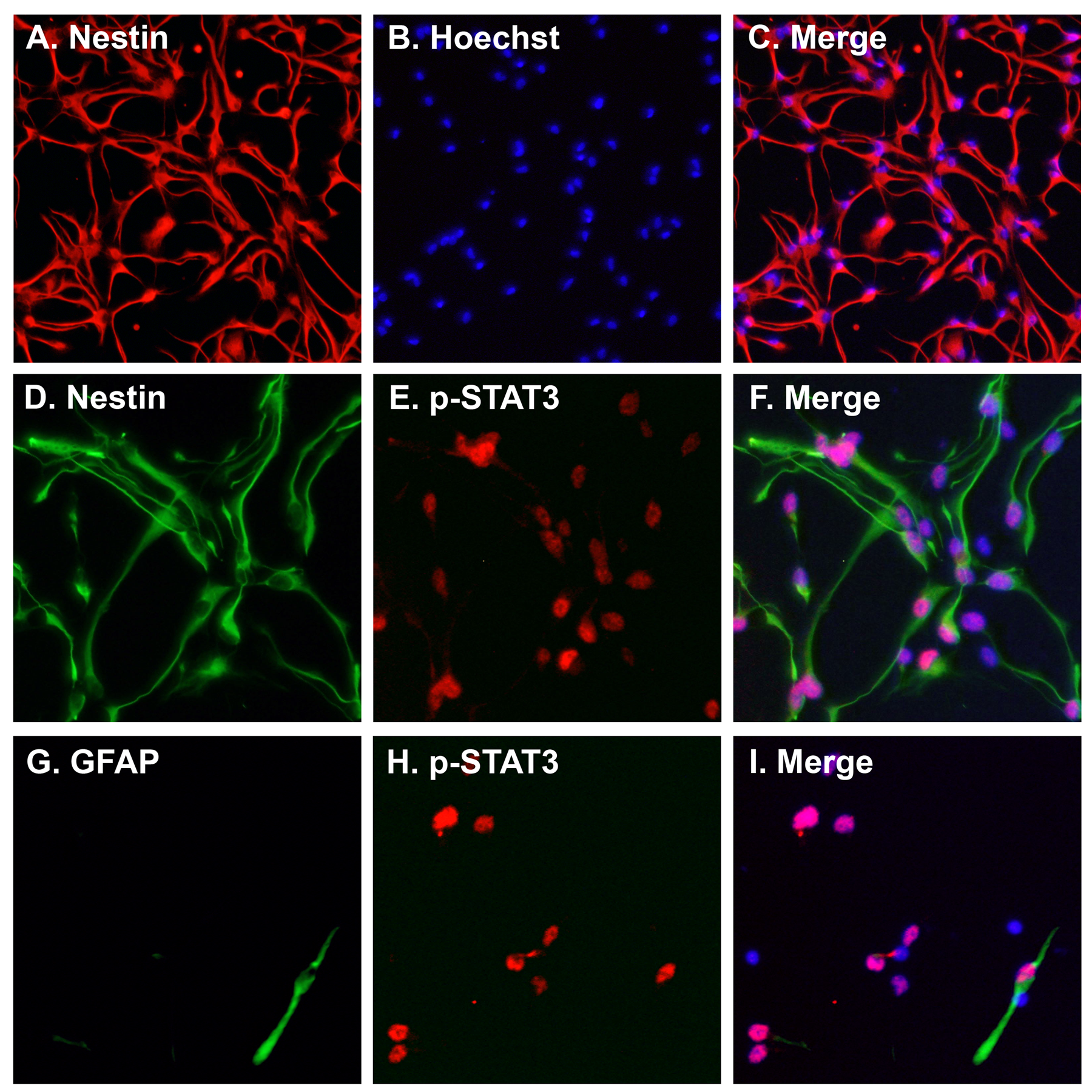

Supplement: Figure S1 — Characterization of human cortical NPCs. A–C. Human fetal cortical NPCs were expanded as neurosphere in NPIM. Cells were dissociated and plated on poly-D-lysine-coated cover slips for 24 h. Cells were fixed and stained for Nestin (red, A). Nuclei were stained using Hoechst 33342 (blue, B). C shows merge of A and B. Original magnification is 20 ×. Results are representative of two donors. D–I. NPCs were cultured in NPIM overnight and were then treated with 20% LPS+HIV MCM in NB27 for 24 h. Cells were immunolabeled with antibodies to phospho-STAT3 (p-STAT3, red, E, F, H and I) and Nestin (green, D and F) or astrocyte marker GFAP (green, G and I). Nuclei were stained with Hoechst (blue in merged pictures F and I). Results are representative of two independent experiments. Original magnification is 40 ×. (TIF) [file pone.0019439.s001.tif]

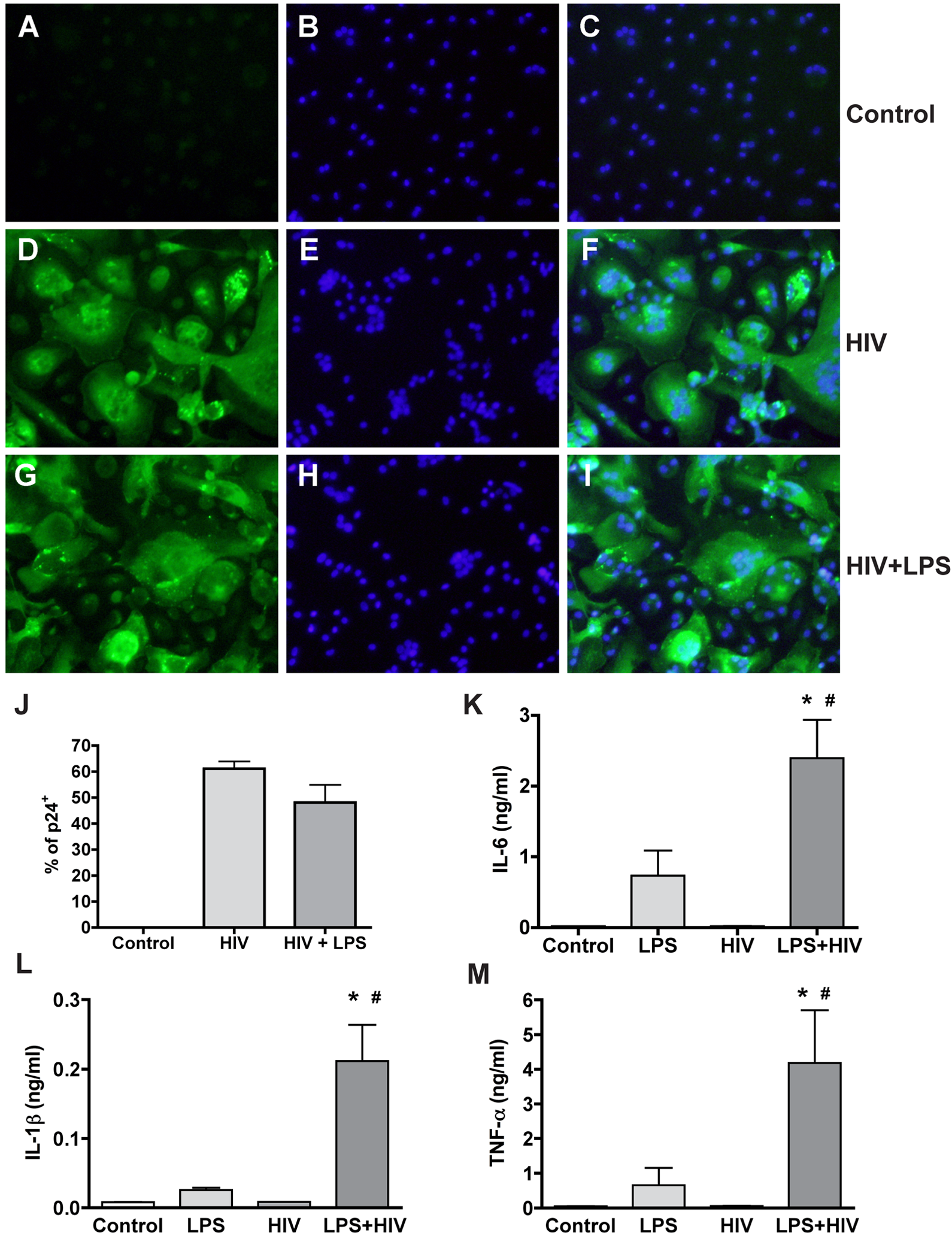

Supplement: Figure S2 — LPS-activated and/or HIV-1-infected MDM induce cytokine production. A–I. HIV-1 infection. MDM were infected with HIV-1ADA for 3–4 days and then stimulated with LPS (100 ng/ml) for 3 h. Cells were stained with antibody to p24 (HIV-1 infection marker, green), conjugated with anti-mouse Alexa fluo 488 nm secondary antibody. Hoechst 33342 was used for nuclear staining. A–C show control uninfected MDM. D–F show HIV-1-infected MDM (HIV). G-I show LPS-activated and HIV-1-infected MDM (HIV+LPS). Panels are representative of three separate donors. Original magnification is 20 ×. J. HIV-1 infection was quantified by determining the percentage of p24-positive cells of seven to ten random microscopy fields. Data is presented as the mean ± SEM. K–M. HIV-infected and/or LPS-activated MCM were collected and measured for levels of IL-6 (K), IL-1β (L), and TNF-α (M) by ELISA. Data is presented as the mean ± SD. Results represent the average of four donors. * p<0.001 in comparison to con-MCM, # p<0.001 in comparison to LPS-MCM. (TIF) [file pone.0019439.s002.tif]
